# Draft Genome Sequence of *Rhodococcus erythropolis* JCM 6824, an Aurachin RE Antibiotic Producer

**DOI:** 10.1128/genomeA.01026-14

**Published:** 2014-10-09

**Authors:** Wataru Kitagawa, Miyako Hata, Tsuyoshi Sekizuka, Makoto Kuroda, Jun Ishikawa

**Affiliations:** a Bioproduction Research Institute, National Institute of Advanced Industrial Science and Technology (AIST), Sapporo, Japan; b Graduate School of Agriculture, Hokkaido University, Hokkaido, Japan; c Pathogen Genomics Center, National Institute of Infectious Diseases, Tokyo, Japan; d Department of Bioactive Molecules, National Institute of Infectious Diseases, Tokyo, Japan

## Abstract

*Rhodococcus erythropolis* JCM 6824 is the producer of the quinoline antibiotic aurachin RE. This bacterium also degrades and utilizes some aromatic compounds, such as biphenyl and benzoate. Here, we report the draft genome sequence of this strain.

## GENOME ANNOUNCEMENT

Members of the genus *Rhodococcus* are well known for their prominent ability to degrade or utilize diverse recalcitrant compounds, especially aromatics, such as biphenyls, dioxins, and nitrophenols (1, 2). In terms of this, the number of genes involved in degradation and the whole-genomic DNA sequence of *Rhodococcus* species have been analyzed (3–5). In addition to the favorable characteristics, we have demonstrated that members of the genus *Rhodococcus* are prospective antibiotic producers (6, 7). To date, at least 19 strains of *Rhodococcus* have been shown to have antibiotic-producing properties (6, 8). Of these strains, *Rhodococcus erythropolis* JCM 6824, which was originally isolated as a cholesterol-degrading microorganism (9), produces a quinoline antibiotic, aurachin RE (7). Aurachins are potent antibiotic compounds that exert strong activity against Gram-positive bacteria (7, 10, 11). Although a few antibiotic peptides have been reported so far (12, 13), aurachin RE is the first example of a second metabolism antibiotic isolated from rhodococci. A biosynthesis gene cluster (*rau* genes) of the compound was also identified and investigated in strain JCM 6824 (14). However, none of the genomic DNA sequences of antibiotic-producing rhodococci have been reported to date. To better understand the antibiotic production and other abilities, draft genome sequence analysis of *R. erythropolis* JCM 6824 was performed.

The genome of *R. erythropolis* JCM 6824 was determined using the Illumina GAIIx paired-end technology provided by the Pathogen Genomics Center, National Institute of Infectious Diseases (Tokyo, Japan). This sequencing run yielded 22,830,367 high-quality filtered reads, with 80-bp paired-end sequencing, providing approximately 200× genome coverage. The genome was assembled using the Velvet assembler version 1.1.05 (15). The final assembly consists of 198 scaffolds of 284 contigs containing 7,023,610 bp, with 62.3% G+C content and an *N*_50_ length of 407,696 bp. The prediction of protein-coding sequences (CDS) and annotation were performed by the Microbial Genome Annotation Pipeline (http://www.migap.org/), which utilizes MetaGeneAnnotator (16), RNAmmer (17), tRNAscan-SE (18), and BLAST (19).

The draft genome sequence of strain JCM 6824 contains 6,718 putative CDSs, 51 tRNAs, and 3 rRNAs. It also contains 18 copies of the putative cytochrome P450 gene, in addition to one gene that was found in the *rau* gene cluster (14, 20). Additionally, it contains putative second metabolism biosynthesis gene clusters, such as nonribosomal peptide synthetase (10 copies), polyketide synthetase (2 copies), and terpene synthetase (2 copies). In addition, it also contains 7 probable aromatic ring-hydroxylating dioxygenase genes and 6 cholesterol oxidase genes, which were estimated to be involved in the initial step of the degradation of these compounds. This genome information may help to understand the function of biosynthesis of second metabolites and also the biodegradation of aromatics and cholesterols in this strain.

### Nucleotide sequence accession numbers.

The draft genome sequence has been deposited at DDBJ/EMBL/GenBank under accession numbers DF836092 to DF836289. The whole-genome shotgun master numbers are BBLL01000001 to BBLL01000284.
